# Genomic Instability of G-Quadruplex Sequences in *Escherichia coli*: Roles of DinG, RecG, and RecQ Helicases

**DOI:** 10.3390/genes14091720

**Published:** 2023-08-29

**Authors:** Virali J. Parekh, Grzegorz Węgrzyn, Véronique Arluison, Richard R. Sinden

**Affiliations:** 1 Laboratory of DNA Structure and Mutagenesis, Department of Chemistry, Biology and Health Sciences, South Dakota School of Mines and Technology, Rapid City, SD 57701, USA; virali.parekh@mines.sdsmt.edu; 2 Department of Molecular Biology, University of Gdansk, Wita Stwosza 59, 80-308 Gdansk, Poland; grzegorz.wegrzyn@biol.ug.edu.pl; 3 Laboratoire Léon Brillouin LLB, CEA, CNRS UMR12, CEA Saclay, 91191 Gif-sur-Yvette, France; 4 UFR Sciences du Vivant, Université Paris Cité, 75006 Paris, France

**Keywords:** DNA repeat, genomic instability, alternative DNA structure, replication slippage, quadruplex, mutagenesis, antigenic variation, bacterial chromatin, helicase

## Abstract

Guanine-rich DNA can fold into highly stable four-stranded DNA structures called G-quadruplexes (G4). Originally identified in sequences from telomeres and oncogene promoters, they can alter DNA metabolism. Indeed, G4-forming sequences represent obstacles for the DNA polymerase, with important consequences for cell life as they may lead to genomic instability. To understand their role in bacterial genomic instability, different G-quadruplex-forming repeats were cloned into an *Escherichia coli* genetic system that reports frameshifts and complete or partial deletions of the repeat when the G-tract comprises either the leading or lagging template strand during replication. These repeats formed stable G-quadruplexes in single-stranded DNA but not naturally supercoiled double-stranded DNA. Nevertheless, transcription promoted G-quadruplex formation in the resulting R-loop for (G_3_T)_4_ and (G_3_T)_8_ repeats. Depending on genetic background and sequence propensity for structure formation, mutation rates varied by five orders of magnitude. Furthermore, while in vitro approaches have shown that bacterial helicases can resolve G4, it is still unclear whether G4 unwinding is important in vivo. Here, we show that a mutation in *recG* decreased mutation rates, while deficiencies in the structure-specific helicases DinG and RecQ increased mutation rates. These results suggest that G-quadruplex formation promotes genetic instability in bacteria and that helicases play an important role in controlling this process in vivo.

## 1. Introduction

Repeated DNA motifs are common in genomes and can adopt noncanonical B-form conformations. These include hairpins, cruciform, H-DNA, or triplex structures [1,2,3,4]. Among these conformations, a guanine quadruplex (G4) can form a planar assembly of G’s through Hoogsteen hydrogen bonds [5]. Successive G-planes stack on top of each other and form a structure that may be stabilized by the presence of a cation [6]. G4 usually forms from four tracts of at least three guanines [7,8,9]. The topology of these stacking quadruplexes is highly variable, and strands can be arranged in a parallel, antiparallel, or mixed orientation [10]. A single repeat motif may even form multiple structures depending on the ionic conditions [11,12].

Sequences prone to G-quadruplex formation are found throughout all genomes, from archaea to higher eukaryotes. Although G4s were originally identified in eukaryotes telomeres, oncogene promoters, or the DNA region coding for immunoglobulin heavy-chain switch regions, they are also present in bacteria. G4 sequence patterns were mapped in the *Escherichia coli* genome and found more than 3000 times [13]. In eukaryotes, G4-forming regions often correlate with genomic regions that are unstable, such as in many oncogene promoters, where they could participate in the regulation and expression of these genes [9,14,15,16]. Furthermore, the expression of many genes is influenced by mutations affecting the functions of the RecQ family of helicases, mainly the Bloom syndrome protein (BLM) and the Werner syndrome protein (WRN) helicases that can unwind quadruplexes. This suggests a potential global role for G4-helicases in gene expression regulation [17]. For this reason, the association of G-quadruplex sequences with oncogenes and telomeres has been a focus for the development of therapeutic intervention [18].

Note that in addition to G4 sequences, on the complementary strand of G-quadruplexes, C-rich sequences can also form a four-stranded motif called an *i-motif* [19,20]. In this motif, cytosines form two interdigitated C•C^+^ base-paired strands at a low pH [21], as shown in human telomere repeats and several oncogene promoters.

Since G-quadruplexes can be very stable, they can result in DNA replication pausing in vivo. A replication fork blockage requires a subsequent restart pathway, and mutations can occur during this process [22,23]. Thus, DNA helicases that unwind replication-blocking structures, including G-quadruplexes, and can influence genomic instability have been identified [24]. Additionally, proteins other than helicases (ATP independent) may also influence the genetic instability in bacteria [25,26,27]. Therefore, the enzymes involved in replication restart and recombination may be expected to influence the instability of G-quadruplex-forming repeats. One might expect these proteins to be potential therapeutic targets [28].

In this work, we focused our analysis on the role of three helicases in resolving G4 in vivo. Note that we will not discuss the role of *E. coli* Rep helicase, a protein serving during DNA replication and implicated in resolving *E. coli* DNA G4, as this has been analyzed previously [27,29]. Moreover, although G4 can be formed in RNA strands and despite the fact that G-quadruplexes might influence translation efficiency, we will not consider these processes as we focused on genetic instability at the level of chromosomal DNA (through monitoring specific deletions).

In *Escherichia coli*, several helicases and nucleic acid remodeling proteins could potentially affect G-quadruplexes [26]. Few bacterial G4 helicases have been identified, but the best characterized is bacterial RecQ. RecQ belongs to a conserved family of helicases important for genome maintenance [30] (see above, WRM and BLM). The RecQ family of helicases can unwind G-quadruplex structures [31,32]. Nevertheless, while in vitro approaches have shown that bacterial RecQ can resolve G4s, it is unclear whether it plays a role in vivo [27]. Recently, a structure of the RecQ bound to G4 DNA was resolved [33], evidence of a guanine-flipping and sequestration model.

Another helicase that may play a role in G-quadruplex stability is that encoded by the *dinG* gene. DinG unwinds branched DNA structures from 5′ to 3′ [34,35]. DinG requires a 5′ single-stranded tail of 11–15 nucleotides, and it unwinds both D-loops and R-loops [34]. D-loops and R-loops are comprised of a single-stranded bubble displaced by a DNA or RNA strand hybridized to the complementary strand [36]. DinG is a homolog of the human protein FANCJ that can unwind G4s. DinG and Rep may cooperate to promote G4 unwinding [37].

Finally, RecG is a structure-specific, branched-DNA helicase (translocase) involved in recombination during DNA replication restart [38]. RecG has also been shown to resolve four-stranded DNA structures [38]. Nevertheless, its possible role in resolving G4 in vivo has not been addressed previously.

In this work, to understand the relationships between G-quadruplex formation in cells, helicases, and genomic instability, G-quadruplex forming repeats with different propensities for formation were cloned into the chloramphenicol acetyltransferase gene (*cat*) in the plasmid pBR325. This genetic system, operating in *E. coli* cells, reports complete or partial deletion and frameshift mutations, as well as differences due to the location of the G-quadruplex-forming sequence in either the leading or lagging template strand of replication [39]. The analysis of the mutation rates can provide information about the relationship between G-quadruplex formation in cells and genetic instability. Here, we examined the influence of DinG, RecG, and RecQ helicases on bacterial G4-genetic instability.

## 2. Materials and Methods

### 2.1. Bacterial Strains and Media

The *E. coli* strains used were HB101 (*supE44 hsdS20*(r_B_^-^m_B_^-^) *recA13 ara-14 proA2 lacY1 galK2 rpsL20 xyl-5 mtl*), BW25113 (Δ(araD-araB)567, ΔlacZ4787::rrnB-3 λ^-^ *rph-1* Δ(*rhaD-rhaB*)*568 hsdR514*), JW0784 (BW25113 with Δ*dinG771*::*kan*), AB1157 (F^-^ *thr-1 leuB6 thi-1 lacY1 galK2 ara14 xyl5 mtl-1 proA2 his4 argE3 rpsl3* (Str^R^) *tsx-33 supE44 kdgK51*), DL1104 (AB1157 with Δ*recG263*::*kan*), SWM1003 (AB1157 with Δ*recQ*::*kan*), and N6916 (AB1157 with Δ*recG263*::*kan* Δ*recQ*::*kan*).

K media and LB (Luria-Bertani) broth have been described [23,40] and were supplemented, when needed, with ampicillin at 30 μg/mL. K medium diluted with M9, salts, and thiamine but without glucose or casamino acids was used for some mutation rate assays. To select Cm-resistant (Cm^r^) bacteria, 25 μg/mL of chloramphenicol (Cm) was added to the LB plates.

### 2.2. Cloning Quadruplex-Forming Repeats

Complementary oligonucleotides for (G_3_T)_4_, (G_3_T)_8_, (G_3_T_2_)_4_, (G_4_T)_4_, (G_4_T_2_)_4_, and RET G4 DNA inserts were obtained from Integrated DNA Technologies, Coralville, IA, USA. For structural studies, complementary oligonucleotides were hybridized and then ligated in the EcoRI site of pGEM^®^-Z3 (Promega, Madison, WI, USA). For mutation rate assays, DNA inserts were cloned into pBR325 [40,41] and confirmed by sequencing. The RET G4 repeat [(G_4_C)_3_G_5_C] was 30-bp but contained an in-frame TAG stop codon. This design, in one orientation, should favor the selection of predominantly complete deletions between the flanking EcoRI sites. pBR235 plasmids contained a revered BsrBI fragment, constructed as described in [25]. Plasmids were purified for structural studies from ethidium bromide CsCl (Sigma, Saint Louis, MO, USA) density gradients.

### 2.3. Mutation Rate Measurement and Analysis of Cm^r^ Revertants

Luria-Delbrück fluctuation analyses were performed to determine the mutation rates [42]. Eighteen parallel 1-mL *E. coli* cultures (started at ~10^4^ cells/mL) were grown from overnight cultures of a single colony. Viable cell counts were an average of three independent cultures plated on LB-ampicillin plates. Cm^r^ revertants were determined from LB-Cm plates. The MSS estimator [43] was used to calculate mutation rates.

Due to different inherent mutation rates, different media were used for some of the bacterial strains. The inserts with the highest mutation rates had rates too high for analysis using 1 mL cultures of LB or K medium. By limiting glucose and casamino acids (CAA), the final cell density could be adjusted to result in a number of Cm^r^ colonies that could be accurately counted (~200–400). These analyses were performed using 150 μL cultures grown in a 96-well microtiter dish. For strains BW25113 and JW0784, K medium was used for cells containing plasmids with (G_3_T)_4_, 0.001 K medium (0.001% glucose and CAA) for (G_3_T)_8_, 0.01 K medium (0.01% glucose and CAA) for (G_3_T_2_)_4_, 0.025 K medium (0.025% glucose and CAA) for (G_3_T)_4_-dimer, 0.001 K medium for (G_4_T)_4_, 0.2 K medium (0.2% glucose and CAA) for (G_4_T_2_)_4_, and 0.001 K medium for G_4_T-dimer. For AB1157 and derivatives, 0.005 K medium (0.005% glucose and CAA) was used for (G_4_T)_4_-dimer, and 0.005 K medium for (G_3_T)_8_. Mutation spectra were determined as described [25,40].

## 3. Results

### 3.1. Confirmation of the DNA Structure Formation in G-Quadruplex-Forming DNA Repeats

(G_3_T)_4_, (G_4_T)_4_, (G_3_T_2_)_4_, (G_4_T_2_)_4_, (G_3_T)_8_, and the RET oncogene repeat (G_4_C)_3_G_5_C form G-quadruplex structures that have been well characterized structurally [25]. Circular Dichroism (CD) has, for instance, been used to confirm they adopt a specific quadruplex conformation [25,44]. DNA oligonucleotides with (G_3_T)n and (G_4_T)n repeats formed parallel quadruplexes with a CD peak at 264 nm and a minimum at 243 nm [44]. The Ret G4 repeat also gave a spectra diagnostic for a parallel strand quadruplex, as reported previously [45]. The CD spectra of (G_4_T_2_)_4_ in NaCl and KCl exhibited peaks at 265 nm and ~292 nm, with a minimum at ~243 nm, which is diagnostic for a 3 + 1 structure [44]. Note that the (G_3_T_2_)_4_ repeat formed a 3 + 1 G-quadruplex in NaCl but a parallel G-quadruplex in KCl. Finally, the complementary C-rich strand could form an i-motif. The (C_3_A)_4_ repeat exhibited a CD peak at ~290 nm at pH 4.7, diagnostic for an i-motif, while the spectra at neutral pH were characteristic of single-stranded DNA [44]. All the spectra can be found in and downloaded from our Nucleic Acid Circular Dichroism Database NACDDB https://genesilico.pl/nacddb/ (accessed on 28 August 2023) [44]. Relative stabilities (Tm) of the various structures are given in Supplementary Table S1.

### 3.2. Plasmids for the Analysis of the Genetic Instability of G-Quadruplex-Forming Repeats

To understand the inherent genetic instability of G-quadruplex-forming DNA repeats, the various sequences were cloned in the EcoRI site of the plasmid pBR325 (Figure S1). As the stability of G-quadruplex structures varies with the sequence, this allows for the analysis of the correlation of inherent stability with the rates of instability. Insertion in the EcoRI site in the gene encoding chloramphenicol acetyltransferase (*cat*) renders cells chloramphenicol sensitive (Cm^s^). Genetic instability within the G-quadruplex-forming repeats, resulting in complete deletion between flanking EcoRI sites, partial deletion, or simple frameshift mutation, can result in a chloramphenicol-resistant (Cm^r^) phenotype, as observed in other repeats [40]. To ascertain the genetic instability differences when a G-rich sequence resides in the leading or lagging replication strands, the ampicillin-resistance (*bla*) gene and unidirectional replication origin ColE1 were reversed for the pBR235 series plasmids [25].

### 3.3. Measurement of the Mutation Rates of Quadruplex-Forming Repeats in pBR325 and pBR235

**Figure 1:**
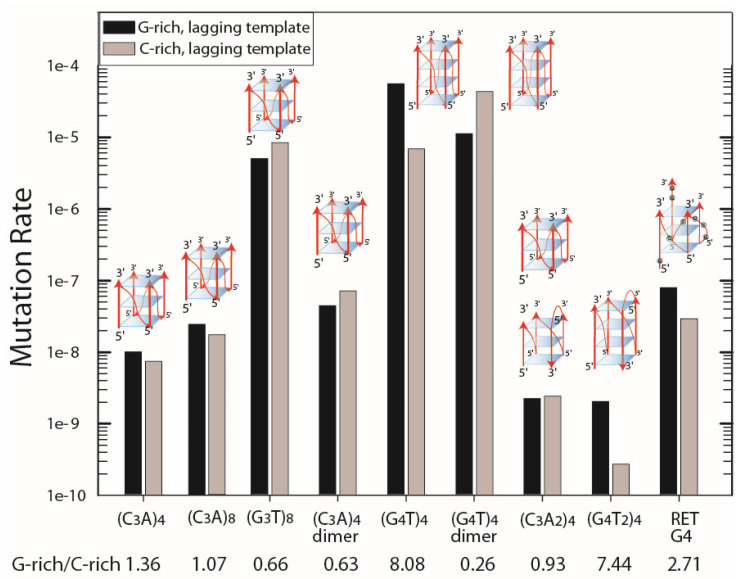
Mutation rates in BW25113. The results are plotted for the G-rich or C-rich tract comprising the lagging template strand during replication. Black bar, G-rich strand in the lagging template; grey bar, C-rich strand in the lagging template. G-rich/C-rich is the ratio of mutation rates. Images of minimal G4 structures are shown above the data that correspond to the different G4 structures that can form in the G-rich strands (also see Supplemental Table S1). Note that (G_3_T)_8_ can form one or two G4 structures, as can dimer repeat inserts.

Chloramphenicol resistance (Cm^r^) mutation rates for G-quadruplex-forming repeats were measured in strain BW25113 (wild type), as described in Materials and Methods. In this genetic background, mutation rates varied by more than 10^5^ as a function of the DNA sequence, ranging from 5.5 × 10^−5^ to 2.7 × 10^−10^ Cm^r^ revertants per cell per generation (Figure 1). In the three repeats containing (C_3_A)_n_, longer repeats showed higher rates of instability, with (C_3_A)_4_-dimer > (C_3_A)_8_ > (C_3_A)_4_. The large increase in mutation rates (factors of 270 and 354) between (C_3_T)_8_ and (G_3_T)_8_ reflected the possibility of transcription-driven G4 formation when the G-rich strand comprised the non-template strand, as discussed previously [25]. The RET G_4_ repeat, which can also form a stable parallel G-quadruplex, had a mutation rate similar to that of the (C_3_A)_4_-dimer repeat. The two plasmids containing (G_4_T)_4_ ((G_4_T)_4_ and (G_4_T)_4_-dimer) that formed a parallel G-quadruplex in vitro also exhibited high rates of Cm^r^ reversion. Repeats with a TT dinucleotide interspersed between G3 or G4 tracts, which form less-stable structures, including the 3 + 1 G-quadruplex in NaCl, exhibited the lowest rates of Cm^r^ reversion.

Mutation rates varied from 0.26-fold to 8.08-fold, dependent on the orientation of the replication origin ColE1 (Figure 1). Mutation rates were 8.08-fold, 7.44-fold, and 2.71-fold higher for (G_4_T)_4_, (G_4_T_2_)_4_, and RET G4 repeats, respectively, when the G-rich strand comprised the lagging template. The mutation rate for the (G_4_T)_4_-dimer repeat was 4-fold higher when the G-rich strand comprised the leading template.

Note that the use of scramble sequences to analyze the mutation rate imposed by the genetic state of the host strain, regardless of the sequence with this system, has been evaluated in our previous works [39,40,46,47,48,49]. We concluded from these works that the Cm^r^ reversion frequencies for this series of “scrambled rate” inserts ranged from 10^−10^ to 10^−9^. Also, note that ~10^−10^ approximates the limits of detection in this genetic selection test and that sequences here that are less prone to structure formation (C_3_A_2_)_4_, and (G_4_T_2_)_4_ (Supplementary Table S1) has low mutation rates approaching this lower limit of detection.

### 3.4. Influence of DinG on the Genetic Instability of Quadruplex-Forming Repeats

To ascertain the involvement of the DinG helicase on G-quadruplex repeat instability, mutation rates were measured in the *dinG* mutant and isogenic *dinG*^+^ parental strains for both orientations of the replication origin. The mutation rates for plasmids in BW25113 (wild type) and JW0784 (Δ*dinG*) are shown in Table 1. For the (G3T)_4_ and (G3T)_8_ repeats, in both orientations, the mutation in *dinG* resulted in a 3-fold and ~7-fold increase in mutation rates, respectively. Other repeats analyzed showed smaller effects (<2-fold).

### 3.5. Influence of RecG and RecQ Helicases on the Instability of Quadruplex-Forming Repeats

The (G_3_T)_8_ and (G_4_T)_4_-dimer repeats were analyzed in AB1157 background strains containing mutations in the *recG* and *recQ* genes, encoding RecG or RecQ helicases, respectively, and in a *recG recQ* double mutant. For the (G_3_T)_8_ repeat, the mutation rates ranged from 5.95 × 10^−9^ to 1.39 × 10^−8^ in pBR325 when the G-rich tract comprised the leading template and from 3.12 × 10^−8^ to 1.18 × 10^−6^ in pBR235 when the G-rich tract comprised the lagging template (Table 2). In this genetic background, a significant dependence on orientation for mutation rates of 5-fold to 174-fold was observed, with a higher mutation rate when the G-rich strand constituted the lagging template.

The role of the RecG and RecQ helicases in the instability of G-quadruplex-forming repeats was assessed by comparing the mutation rates in wild type and mutant strains. For the (G_3_T)_8_ repeat, little effect was observed for the stable orientation (pBR325) when the G-rich strand comprised the leading template (C-rich, lagging strand) (Table 2). In contrast, when the G-rich strand comprised the lagging template, the *recG* mutation resulted in a 3.4-fold decrease in mutation rate, while the *recQ* mutation resulted in a 1.95-fold increase in this rate. The *recG recQ* double mutant exhibited a 10.8-fold increase in the mutation rate. Cm^r^ reversion rates for strains containing the (G_4_T)_4_-dimer, which, as discussed below, may not form alternative structures in cells, showed less than 2-fold differences in the *recG, recQ*, and *recG recQ* mutant strains compared with the parental strain for both orientations (Table 2). This repeat did show leading/lagging asymmetries with a higher rate when the G-rich strand comprised the lagging template.

### 3.6. Cm^r^ Mutation Spectra for E. coli Containing Quadruplex-Forming Repeats

Cm^r^ revertants were scored for complete or partial deletions using PCR and DNA sequencing, as described previously [25,40]. Different repeats underwent different types of mutation in the *cat* gene, and this was generally independent of genetic background, although some effects of the specific mutations analyzed were observed.

For the (G_3_T) series repeats that form the most stable G-quadruplex, different mutation spectra were observed for the 3 repeats. The (G_3_T)_4_-dimer underwent complete deletions between EcoRI sites in 24 of 25 revertants analyzed, with one partial deletion to a 21-bp insert. The (G_3_T)_8_ repeat predominantly exhibited deletion of (G_3_T)*_2_*, leaving a 30-bp insert with the remaining events being complete deletions. In the combined BW25113 and AB1157 backgrounds, 59/62 and 44/48 events were a (G_3_T)_2_ deletion in pBR325 and pBR235, respectively. For the (G_3_T)_4_ repeat, when the G-rich strand comprised the lagging template, 67% (6/9) and 100% (10/10) complete deletions occurred in the wild type and *dinG* mutant cells, respectively. In the opposite orientation, 56% (9/16) and 60% (6/10) complete deletions occurred in the wild type and dinG mutant cells, respectively.

For the (G_4_T) series repeats, complete deletions occurred for the (G_4_T)_4_-dimer in 85% (39/47) of the revertants analyzed in the BW25113 background and 97% (70/72) in the AB1157 derivatives. The (G4T)_4_ repeat underwent deletion of one (G4T) repeat (at a high rate) in all cases examined (46/46).

For the (G_3_T_2_)_4_ and (G_4_T_2_)_4_ repeats that form the less stable 3 + 1 G-quadruplex and were the most stable genetically, partial deletions occurred preferentially (90–100% in pBR325 and 60–62% in pBR235 plasmids). The deletion mutations analyzed were all consistent with replication slippage. The sequences of major and representative mutations are shown in Figure S2.

## 4. Discussion

G-quadruplex-forming DNA repeats occur upstream of many human promoters, including those of proto-oncogenes. The correspondence of breaks and chromosomal rearrangements associated with these sequences is consistent with an interpretation that the formation of an alternative DNA structure, specifically a G-quadruplex in duplex DNA, may lead to replication fork blockage and ultimately a double-stranded break that may initiate events responsible for genomic instability [23]. To understand how G-quadruplex structure formation in cells can lead to bacterial genomic instability, we have characterized the relative genetic instability of a series of G-quadruplex-forming repeats in an *E. coli* model mutagenesis system. The rates of genomic instability and reversion to chloramphenicol resistance by complete or partial deletion are consistent with the interpretation that, for certain repeats, G-quadruplex formation during transcription, replication, or replication restart may promote the genetic instability of the repeats. In addition, the DinG, RecG, and RecQ helicases influence rates of genetic instability associated with these repeats.

Structural analysis, stability, and rate of formation measurements in single-stranded DNA have been completed for simple G-quadruplex forming sequences, including those analyzed here [50]. In general, an increase in loop length decreases the stability of the G-quadruplex [51].

**Figure 2:**
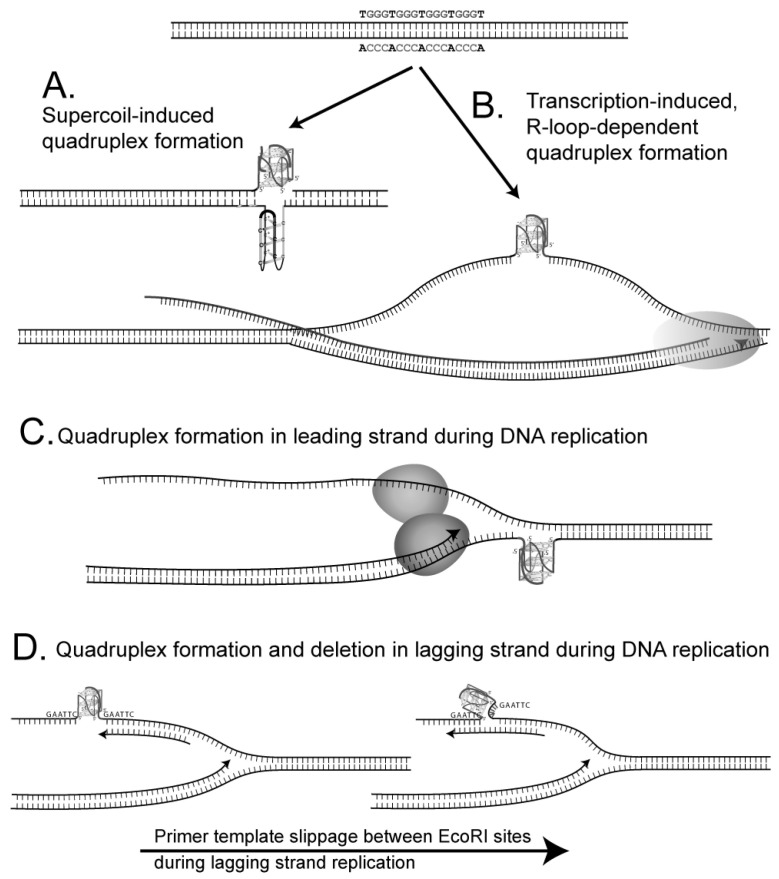
Pathways to quadruplex formation and deletion. Quadruplex structures may form spontaneously in supercoiled DNA (**A**) in a displaced strand following R-loop formation (**B**) in the leading strand ahead upstream of a replication fork (if helicases unwind the strands well ahead of the fork (**C**) or in a single-stranded lagging template strand of replication (**D**). In (**D**), complete deletion likely occurs by replication slippage that involves primer-template misalignment between the two proximal EcoRI restriction sites generated when repeats are cloned into the *cat* gene. A stable G-quadruplex would be expected to block replication (left), thereby promoting replication slippage (right). This is shown in the context of lagging strand replication, although it could happen during replication of either strand.

G-quadruplex structures may form in cells by different pathways (Figure 2). First, a G-quadruplex may form driven by the energy inherent in negatively supercoiled DNA. An i-motif may then form in the complementary strand (Figure 2A). Noncanonical structures may form following strand denaturation and slow renaturation [52] or potentially during incubation in a buffer containing K^+^ that acts to stabilize G-quadruplex structures [53]. Our extensive analyses failed to observe a structural transition in supercoiled DNA in a physiological ionic strength buffer for any of the G-quadruplex-forming repeats studied here, consistent with Sekibo and Fox [54]. Transcription resulting in a G-rich RNA strand can cause the formation of an R-loop, leading to a G-quadruplex in the displaced strand (see Figure 2B), as shown by Duquette et al. and Parekh et al. [25,55,56]. The formation of an RNA-DNA hybrid during the transcription of a G-rich tract appears to be common [57]. 

**Figure 3:**
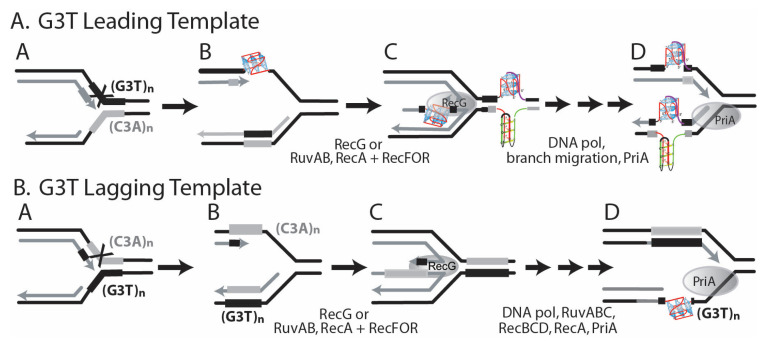
G-quadruplex formation during replication pausing and restart as a source of genetic instability. (**A**) G_3_T Leading Template. (A) Leading strand replication pausing during synthesis of the G-rich strand of (G_3_T)_n_. (B) After pausing, a G-quadruplex may form in the leading template. Replication may continue on the lagging template. (C) RecG-dependent fork reversal could place a G-quadruplex/i-motif ahead of the replication fork. It is not known if DNA repair machinery would work on the G-quadruplex/i-motif structure prior to replication restart. G-quadruplexes are shown in blue, while i-motifs are shown in green. (D) Replication on the ‘chicken foot’ following primer-template misalignment may also encounter a G-quadruplex, which could lead to deletions in the leading template. After branch migration, a G-quadruplex/i-motif could also form in the lagging strand, although it may not block continued fork progression. Note that an alternative restart pathway involving Holliday junction cleavage is not shown (see Kim et al. [23]). (**B**) G_3_T Lagging Template. While similar intermediates can be drawn when the G-rich repeat (G_3_T)_n_ comprises the lagging template, the opportunity for formation of a G-quadruplex within a single strand is limited to late steps of a Holliday junction cleavage, recombination, and replication restart.

Moreover, our results confirmed transcription-driven G-quadruplex formation in the (G_3_T) series repeats in vitro [25]. In cells with the (G_3_T)_8_ repeat in an orientation that supports R-loop formation in vivo, deletion rates are dramatically increased compared with the opposite orientation where R-loop and structure formation do not occur. A replication fork may encounter a G-quadruplex structure once formed within an R-loop. pBR325 and pBR235 would contain the G-quadruplex in the leading and lagging template strands, respectively. Another possibility for G-quadruplex formation involves formation in the lagging strand when it is single-stranded (Figure 2D). As the leading strand generally remains duplex, a lower probability may exist for G-quadruplex formation prior to the arrival of the replication fork (Figure 2C). However, if the helicase were uncoupled from polymerase, a single-stranded region may be generated that could support G-quadruplex formation. If, however, a replication fork blockage occurs at a G-quadruplex-forming repeat during replication due to inherent pausing or an encounter with a DNA secondary structure, then replication restart pathways provide many opportunities in regions of single-stranded DNA for G-quadruplex structure formation, as shown in Figure 3A. If the G-rich repeat comprises the lagging template, replication restart pathways afford fewer opportunities for structure formation (Figure 3B). An additional factor that may influence the probability of formation is that, in pBR325, the direction of replication and transcription are convergent, which may slow or block replication.

Many different mutations within the G-quadruplex region can result in a Cm^r^ phenotype. First, replication slippage between proximal EcoRI sites flanking the G4-forming sequences will result in the complete deletion of the cloned insert, as shown in Figure 2D. Second, replication slippage within a G4-forming repeat tract can restore the reading frame, resulting in a Cm^r^ phenotype. Third, replication slippage that does not involve the G4-forming repeats can restore the reading frame and Cm^r^ phenotype. The rate and type of mutation event observed are often dependent on the secondary DNA structure. Moreover, mutation rates and spectra observed will also reflect the frequency with which an event occurs that leads to a Cm^r^ phenotype. For example, Cm^r^ reversion by a single trinucleotide deletion within (CTG)_25_•(CAG)_25_ can occur at a rate of 5 × 10^−2^, but lower rates in longer repeat tracts reflect the requirement for larger deletions [40].

Complete deletion of palindromic inserts cloned between flanking EcoRI sites can occur at very high frequencies, and this is believed to be associated with a cruciform or hairpin formation [39,46,48]. In addition, imperfect hairpins within (CTG)•(CAG) repeats are believed to promote high frequencies of deletions [40,58]. Moreover, asymmetries in rates of deletions and duplications in leading or lagging replication strands are frequently observed with cloned inserts that can form alternative DNA structures [39,40,46,48,58,59]. A working hypothesis evidences that a G4 structure blocks the replication fork and brings the flanking restriction sites into closer juxtaposition, which then facilitates misalignment between the proximal restriction sites (Figure 2D), and this is responsible for the high frequency of deletion.

Complete deletions were the predominant event for (G_3_T)_4_, (G_3_T)_4_-dimer, and the (G_4_T)_4_-dimer repeats. Complete deletions in (G_3_T)_4_ are consistent with replication slippage across a G-quadruplex. In the *dinG* mutant, when the G-rich tract comprises the lagging template, all revertants showed complete deletions, compared with 67% of the parental wild type strain. This result may be consistent with DinG activity that prevents the accumulation of G4 structures. In the wild type and *dinG* strains, the (G_4_T)_4_-dimer and (G_3_T)_4_-dimer exhibited predominantly, or exclusively, complete deletions that occurred at relatively high frequencies. While G-quadruplex structure formation may occur in these longer repeat tracts, it is also possible that the central palindromic EcoRI site may favor the formation of an antiparallel Hoogsteen base-paired hairpin that promotes replication slippage at a high frequency in a fashion analogous to that observed for (CTG)•(CAG) repeats [40].

All partial deletions that restored the reading frame and resulted in a Cm^r^ phenotype were consistent with primer-template slippage between repeats. In the (G_4_T)_4_ repeat, deletion of a single (G_4_T) repeat, resulting in (G_4_T)_3_, restored the reading frame resulting in reversion to Cm^r^. Complete deletions were not observed. This simple slippage mutation happened at a very high frequency with a higher rate when the G-rich strand comprised the lagging template. Moreover, the absence of an effect of the *dinG* mutation and the inability of this repeat to form G-quadruplex structures on transcription all suggest that slippage is not associated with G-quadruplex structure formation. Remarkably, the simple slippage of a single (G_3_T) repeat, which restores the reading frame in the most stable G-quadruplex-forming sequence (G_3_T)_4_, was only rarely observed; complete deletions were predominant, consistent with structure formation.

The (G_3_T)_8_ repeat showed a high Cm^r^ reversion frequency from deletion involving two (G_3_T) repeats. This long repeat may form stable G4 structures resulting from transcription in cells. Moreover, mutations in the *dinG*, *recQ*, and *recG* genes encoded structure-specific helicases influenced mutation rates, consistent with structure formation. During transcription in vitro, the (G_3_T)_8_ can form one or two G4 structures [25]. The formation of a stable parallel (G_3_T)_4_ may block DNA polymerase, promoting the deletion of two (G_3_T) repeats.

The low rates of Cm^r^ reversion for the (G_3_T_2_)_4_ and (G_4_T_2_)_4_ repeats, the predominance of partial deletions, the low thermal stabilities of their G4 structures (3 + 1 and parallel for (G_3_T_2_)_4_ in K^+^), and the lack of structure formation on transcription may all be consistent with the lack of structure formation in cells.

Complex sequence changes in a single genetic mutation event can be diagnostic of primer-template misalignments that occur during the synthesis of either the leading or the lagging strand [59,60,61,62,63]. All complete and partial deletions shown in Figure S2 could occur by slippage between direct repeats during the replication of either the leading or lagging strand, as denoted by thick underlines with arrowheads facing in both directions. Two sequenced Cm^r^ revertants represented complex mutations that revealed the direction of replication. First, complete deletion of the (G_3_T)_4_ (the most stable G-quadruplex) occurred concomitantly with a C to G change 5′ to the EcoRI site (Figure S2), consistent with replication slippage in the lagging strand (in pBR235) following synthesis of the first GGGT repeat. Second, in several independent (G_4_T)_4_-dimer revertants, concomitant with the deletion of the entire repeat, the sequence 5′CGTATGG3′ 3′ of the EcoRI site was converted to 5′GGGGTGG3′, consistent with replication slippage following synthesis into the second GGGGT repeat during synthesis of the leading strand (in pBR235). In both cases, polymerase paused after synthesis through the first repeat and initiated a misalignment across the repeat. Replication into a structure could promote pausing and misalignment, as is known to occur during the replication of hairpins [64].

In general, the ease of formation and the stability of alternative DNA structures, which include cruciforms, Z-DNA, and intermolecular triplex structures, increase with the length of the structure-forming DNA repeat [1]. Moreover, the genetic instability (measured biochemically) of the direct repeats that form slipped strand structures can increase as a function of the repeat length [65], although in a genetic assay, a simple slippage that results in a Cm^r^ reversion in a short repeat can occur at a higher rate than a structure-dependent reversion event in a long repeat [40,49]. For the quadruplex-forming sequences, in general, longer repeats exhibited higher mutation rates (Figure 2).

DinG, RecG, and RecQ helicases possess unwinding activities on unusual DNA structures. DinG will unwind branched structures, D-loops, and R-loops [34,35]. RecG and RecQ helicase family members, including the WRN and BLM helicases, will unwind G-quadruplex DNA structures [66,67]. These *E. coli* proteins are important in replication restart activities in which branched structures associated with replication forks are removed and resolved by them [34,35]. In addition, DinG and RecQ helicases may act to remove R-loops to avoid ectopic replication initiation [37].

A mutation in *dinG* increased the mutation rates of complete deletions for plasmids containing (G_3_T)_4_ and partial deletions in (G_3_T)_8_, in either orientation with respect to replication fork progression, by factors of 3.3 and 7, respectively. These results suggest that the DinG helicase may be removing G-quadruplex structures formed in either leading or lagging templates during replication. The mutation rates for the (G_3_T)_4_ and (G_3_T)_8_ repeats differed by 1000-fold, presumably reflecting the potential for structure formation, which is clearly higher for the longer repeat. The similar influence of the *dinG* mutation, when the G-rich strand comprised the leading or lagging template strand, may indicate that DinG is actually acting predominantly to remove the G-quadruplex structures formed within transcription-dependent R-loops, as this is replication orientation independent.

Little effect of a mutation in *dinG* was observed with the (G_4_T)_4_ repeats. However, the mutation, a deletion of one (G_4_T), occurred at a very high rate for this repeat. This high rate of replication slippage may be structure-induced, or, if not, it may mask any structure-dependent mutations that may occur at a much lower rate. While the mutation spectra of predominantly complete deletions for the (G_4_T)_4_-dimer are consistent with structure formation, little effect of the *dinG* mutation was observed. It is also possible that a structure formed with the (G_4_T)_4_-dimer repeat was not recognized or resolved by the DinG helicase.

For cells with deficiencies in the RecG and RecQ helicases, a large influence on mutation rates was evidenced for the (G_3_T)_8_ repeat. In strains of this AB1157 genetic background, mutation spectra were indicative of structure-related instabilities (complete or partial deletions). For the (G_3_T)_8_ repeat, the orientation dependence for mutation rates was highest by factors of 5–176 when the G-rich strand comprised the lagging template strand, where structures may be most likely to form. While a deficiency in RecG caused decreased mutation rates by 3-fold, the deficiency in RecQ and the double mutant showed elevated mutation rates (to a factor of 10), consistent with an interpretation that these helicases may participate in structure removal from the lagging strand template. The (G_4_T)_4_-dimer repeat showed a different pattern of response to the RecG and RecQ helicase dysfunctions. First, mutation rates were overall high (10^−6^ to 4 × 10^−5^) and highest when the G-rich strand comprised the leading strand template. This pattern was also observed in the BW25113 background and both the wild type and *dinG* mutant strains. Mutations in *recG* and *recQ* had little effect on (G_4_T)_4_-dimer repeat Cm^r^ mutation rates. The high proportion of complete deletions is suggestive of replication slippage across a secondary structure, as expected for deletion of inverted repeats forming cruciforms and (CTG)•(CAG) repeats forming hairpins [48]. This may suggest the replication fork pausing during replication of these repeats, as observed for CTG, CGG, and GAA repeats [68]. Replication fork pausing would be associated then with the formation of a complex structure that is not susceptible to removal by the helicases studied here.

## 5. Conclusions

In summary, our results support the proposal that G-quadruplex structures form in *E. coli* cells and promote replication misalignment when a replication fork encounters a stable G-quadruplex. These events are influenced by the actions of DNA helicases. Different effects of different helicases on G4 stability in vivo are intriguing, and they might arise from various specificities of particular enzymes that could result in specific actions (and the final results) depending on both DNA structural features and kinetics of biochemical processes (replication, recombination) occurring in certain genome regions. The observed effects could also participate in the rearrangements associated with promoters containing G-quadruplex-forming sequences, which is supported by previous suggestions [55,69,70]. G4 motifs are indeed over-represented in the regulatory regions of bacterial genes, as evidenced by in silico evaluations [13]. The results of this study may also shed new light on the role of G4s in the physiology of pathogenic bacteria, especially as it was demonstrated that G4 results in antigen variation that may help bacteria evade the host immune system [71]. Other features of bacterial pathogenicity may also be affected in this way, especially in the expression of the genes encoding the proteins involved in bacterial virulence or antibiotic resistance or if the replication of plasmids bearing such genes is influenced. In fact, the importance of such regulations in antibiotic resistance has been demonstrated recently [72]. Finally, our analysis may also give new clues in the emerging field of G4 in the mitochondrial genome, as it was shown that in the quadruplex regions of mitochondrial DNA, genetic variation is enhanced [73].

## Figures and Tables

**Table 1:** Effect of a mutation in *dinG* on Cm^r^ mutation rates.

Mutation Rate						
Quadruplex	G-rich Strand Lagging Template			C-rich Strand Lagging Template		
	wt	*dinG*	*dinG*/wt	wt	*dinG*	*dinG*/wt
(G_3_T)_4_	9.93 × 10^−9^	3.27 × 10^−8^	3.29	7.32 × 10^−9^	2.47 × 10^−8^	3.37
(G_3_T)_8_	4.99 × 10^−6^	3.18 × 10^−5^	6.37	8.28 × 10^−6^	5.94 × 10^−5^	7.17
(G_4_T)_4_	5.50 × 10^−5^	5.50 × 10^−5^	1.00	6.81 × 10^−6^	1.04 × 10^−5^	1.53
(G_4_T)_4_ D	1.10 × 10^−5^	4.78 × 10^−6^	0.43	4.27 × 10^−5^	4.00 × 10^−5^	0.94
RET G_4_	2.88 × 10^−8^	5.04 × 10^−8^	1.75	7.80 × 10^−8^	6.58 × 10^−8^	0.84

**Table 2:** Effect of mutations in genes encoding RecG and RecQ on Cm^r^ mutation rates for the (G_3_T)_8_ and (G_4_T)_4_-dimer repeats.

Genotype	Mutation Rate		Ratio	Mutant/wt	
	G-rich Lag	C-rich Lag	G-rich/C-rich	G-rich	C-rich
(G_3_T)_8_ repeat					
wild type	1.09 × 10^−7^	5.95 × 10^−9^	18.34	1	1
recG	3.12 × 10^−8^	6.25 × 10^−9^	5.00	0.29	1.05
recQ	2.12 × 10^−7^	1.39 × 10^−8^	15.23	1.95	2.34
recG recQ	1.17 × 10^−7^	6.79 × 10^−9^	173.63	10.80	1.14
(G_4_T)_4_-dimer repeat					
wild type	2.70 × 10^−5^	1.87 × 10^−6^	14.41	1	1
recG	3.86 × 10^−5^	1.24 × 10^−6^	31.12	1.43	0.66
recQ	1.56 × 10^−5^	2.55 × 10^−6^	6.11	0.58	1.36
recG recQ	1.72 × 10^−5^	2.38 × 10^−6^	7.23	0.64	1.27

## Data Availability

The data that support the findings of this study are available on request from the corresponding authors.

## Supplementary Materials

The following supporting information can be downloaded at: https://www.mdpi.com/article/10.3390/genes14091720/s1, Figure S1: Relationship between (G_4_T)_4_ repeats, transcription, and replication fork direction in pBR325 and pBR235; Figure S2: Deletion mutations consistent with primer-template misalignment during replication; Table S1: G-quadruplex-forming DNA repeats.
