# Supplementary material for: Genomic Instability of G-Quadruplex Sequences in Escherichia coli: Roles of DinG, RecG, and RecQ Helicases
Source: Genes (Basel). 2023 Aug 29;14(9):1720. doi: 10.3390/genes14091720 (PMC10530614; doi:10.3390/genes14091720)
Supplement: Supplementary file 1 [file genes-14-01720-s001.zip › genes-2569353-supplementary.pdf]

Supplemental Figures, Tables and Legends

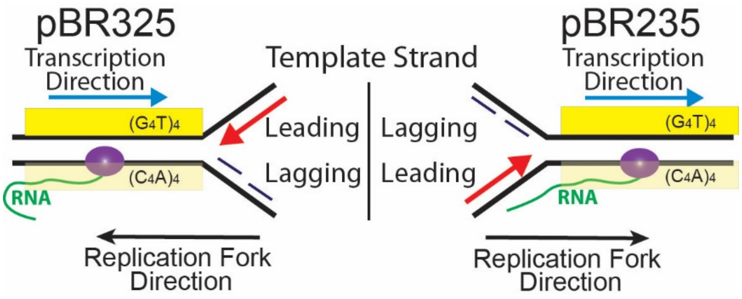

Figure S1. Relationship between $(G_4T)_4$ repeats, transcription, and replication fork direction in pBR325 and pBR235. The unidirectional ColE1 origin of replication is reversed in pBR325 and pBR235, such that the G-rich strand of the $(G_4T)_4$ repeat will comprise the leading or lagging template strands, respectively, when cloned into the chloramphenicol acetyl transferase gene. In pBR325 transcription and the replication fork collide, while in pBR235, transcription and the replication fork are codirectional.

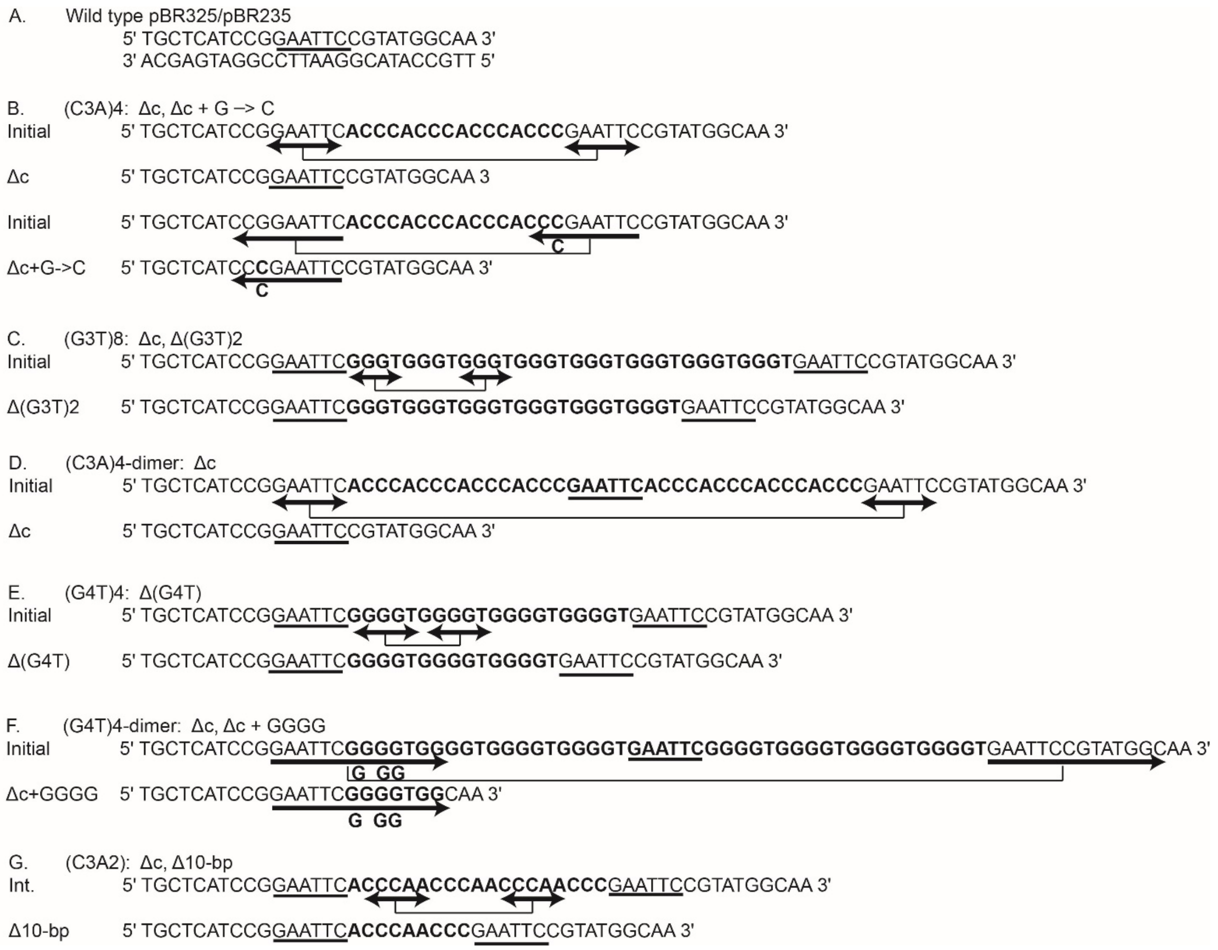

Figure S2. Deletion mutations consistent with primer-template misalignment during replication. A. The sequence around the *Eco*RI site in the CAT gene is shown. B. - G. Initial, shows the sequence of the different G-quadruplex-forming repeats cloned into the *Eco*RI site. The sequence of one or more Cm[r] revertants is then shown. Δ_c, complete deletion. Double headed arrows denote direct repeats, between which primer template misalignment, in either direction of replication, results in deletion. B. $(G_3T)_4$, Δ_c + G→C, arrow denotes misalignment between direct repeats specific for the lagging strand in pBR235 following synthesis of the first GGGT repeat, resulting in deletion with a G to C transversion. C. $(G_3T)_8$, $\Delta(G_3T)_2$, deletion of two (G_3T) units can occur anywhere within the repeat. D. $(G_3T)_4$-dimer. E. $(G_4T)_4$, $\Delta(G_4T)$, deletion of a single (G_4T) can occur anywhere within in the repeat. F. $(G_4T)_4$-dimer, Δ_c+GGGG denotes a complex mutation including complete deletion with 5'CGTATGG3' (3' of the *Eco*RI site) converted to 5'GGGGTGG3' during leading strand replication slippage in pBR235. G. (G_3T_2), Δ10 bp, a 10-bp slippage between repeat units restores the reading frame.

Table S1. Stability of G-quadruplex-forming DNA repeats

Repeat	Sequence	Quadruplex	Tm,℃	
			NaCl	KCl
(G3T)4	gaattc (ACCC)$_4$ gaattc	parallel	61 ± 1	>100℃
(G4T)4	gaattc (GGGGT)$_4$ gaattc	parallel	56 ± 2	97 ± 4
(G3T)8	gaattc (GGGT)$_8$ gaattc	parallel	61 ± 1	>100℃
(G3T2)4	gaattc ACCC(AACCC)$_3$ gaattc	3+1, parallel	40 ± 2	60 ±2
(G4T2)4	gaattc (GGGGTT)$_3$GGGGT gaattc	3+1	50 ± 2	75 ±2
(G3T)4 dimer	gaattc (ACCC)$_4$gaattc(ACCC)$_4$ gaattc	parallel	NA	
(G4T)4 dimer	gaattc (GGGGT)$_4$gaattc(GGGGT)$_4$ gaattc	parallel	NA	
Ret G4	gaattc CGCCCCCGCCCCGCCCCGCCCCTA gaattc	parallel	70 ±1	94 ±4

Tm of the quadruplexes were measured in 10 mM cacodylate, pH 7.0, 100 mM NaCl or KCl. NA, not applicable.
